# The Neural/Immune Gene Ontology: clipping the Gene Ontology for neurological and immunological systems

**DOI:** 10.1186/1471-2105-11-458

**Published:** 2010-09-12

**Authors:** Nophar Geifman, Alon Monsonego, Eitan Rubin

**Affiliations:** 1 Department of Microbiology and Immunology, Faculty of Medical Sciences and The National Institute of Biotechnology in the Negev, Ben Gurion University, Beer Sheva, Israel

## Abstract

**Background:**

The Gene Ontology (GO) is used to describe genes and gene products from many organisms. When used for functional annotation of microarray data, GO is often slimmed by editing so that only higher level terms remain. This practice is designed to improve the summarizing of experimental results by grouping high level terms and the statistical power of GO term enrichment analysis.

Here, we propose a new approach to editing the gene ontology, *clipping*, which is the editing of GO according to biological relevance. Creation of a GO subset by clipping is achieved by removing terms (from all hierarchal levels) if they are not functionally relevant to a given domain of interest. Terms that are located in levels higher to relevant terms are kept, thus, biologically irrelevant terms are only removed if they are not parental to terms that are relevant.

**Results:**

Using this approach, we have created the Neural-Immune Gene Ontology (NIGO) subset of GO directed for neurological and immunological systems. We tested the performance of NIGO in extracting knowledge from microarray experiments by conducting functional analysis and comparing the results to those obtained using the full GO and a generic GO slim. NIGO not only improved the statistical scores given to relevant terms, but was also able to retrieve functionally relevant terms that did not pass statistical cutoffs when using the full GO or the slim subset.

**Conclusions:**

Our results validate the pipeline used to generate NIGO, suggesting it is indeed enriched with terms that are specific to the neural/immune domains. The results suggest that NIGO can enhance the analysis of microarray experiments involving neural and/or immune related systems. They also directly demonstrate the potential such a domain-specific GO has in generating meaningful hypotheses.

## Background

An ontology is a formal way for the representation and sharing of knowledge in a certain domain by describing the concepts (or terms) in that domain and the relationships between them. An ontology formalizes the meaning of concepts, or terms, by a set of assertions and rules that characterize them and connects them to other terms within the ontology [1,2].

The Gene Ontology (GO), a widely used bio-ontology, is used to describe genes and gene products from numerous organisms [3,4]. GO is constructed from three separate ontologies which capture the three main biological areas of knowledge regarding gene products. These ontologies are: molecular function, biological process and cellular component. Thanks to relentless efforts of the Gene Ontology Consortium [5], GO includes a very large number of terms. As of February 2009, it contained approximately 26,800 terms. These terms are interrelated via five relatively simple types of relationship: *is-a, part of*, *regulates*, *positively-regulates *and *negatively-regulates*. Since terms can have more than one parent, the structure of the ontology can be represented as a directed acyclic graph (DAG), in which the terms are the nodes and their relationships are the edges.

One common use of the Gene Ontology is the functional annotation of the results of high throughput experiments, such as transcription profiling arrays [5,6]. In such cases, association is sought between GO terms and genes or gene products that are affected by a particular treatment. Such terms allow the changes in gene expression to be generalized, providing a list of GO terms that characterize the response to a treatment rather than a list of genes.

The fact that GO covers most of biological knowledge related to gene functions may complicate functional analysis. For any given studied system, many terms may be completely unrelated yet are still considered during analysis. This dilutes the number of actual hits, increasing the likelihood of falsely reported enrichment and complicating the interpretation of the resulting list of terms. Consider, for example, the use of the term 'sperm motility' (GO:0030317) when studying systems such as brain aging or eye diseases. Including this term in the enrichment analysis will increase the number of terms considered in the analysis, in turn reducing the statistical power of the enrichment analysis by requiring more rigorous multiple testing correction, while in most likelihood contributing very little to the interpretation of the results.

This problem may be overcome by constructing a domain-specific ontology. Such an ontology can be constructed from scratch by defining all the domain-specific terms and linking them to all those genes that can be defined by these terms. However, this approach is highly demanding and largely overlaps with the massive efforts of the GO consortium.

An alternative approach is to create a subset of GO, choosing terms pertinent to a specific task. Taking this course, existing GO terms (and their relevant relations) are selected from GO rather than defining new terms. This approach is extensively used in generating GO 'slims', which are frequently used for creating a birds-eye view of the results, allowing the results to be summarized and compared [6,7]. Slimming of GO is achieved by choosing high level terms from each of the three major component gene ontologies: cellular component, biological process, and molecular function. The resulting GO slim typically involves a small number of annotations, and separates gene products into very broad categories such as 'metabolism' or 'signaling'. However, while useful for achieving a high level of generalization, the massive loss of resolution greatly reduces the ability of GO-slims to pinpoint relevant processes. A second approach to creating GO subsets uses a method that describes each GO term according to the amount of information it holds. Thus, top-level terms which are more general are assigned a lower 'information score', while more specific terms are assigned a higher score. By describing GO terms in this manner, GO subsets (called partitions) containing terms with consistent information content (i.e., the same level of specificity) can be created [8]. Although this approach allows creation of subsets that contain GO terms at any level of specificity by setting a desired threshold of abstraction, many of these terms may still be biologically irrelevant for the analysis of a specific dataset.

**Figure 1:**
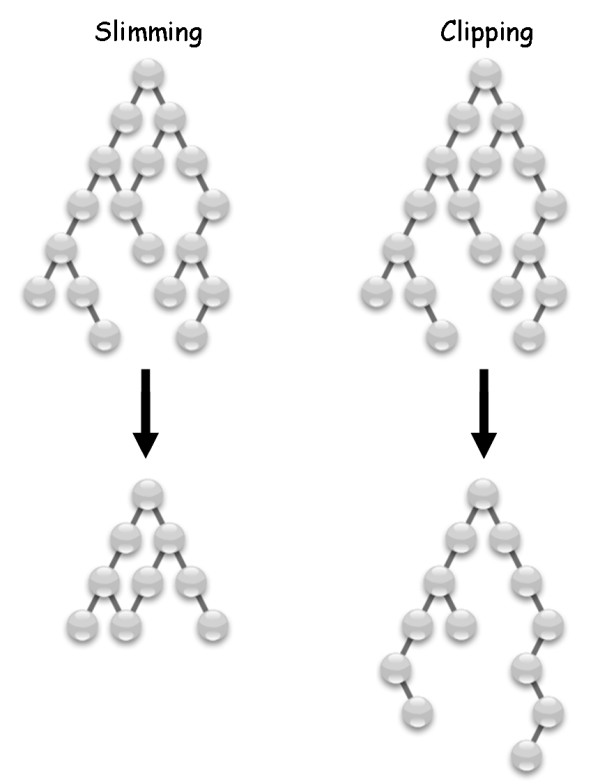
**Comparison of the two methods used for editing the Gene Ontology**. Comparison of the two methods used for editing the Gene Ontology, i.e. slimming and clipping. Slimming of GO involves the assignment of high level terms from each of the three major gene ontologies, namely cellular component, biological process, and molecular function, to a set of genes of interest. Clipping of GO involves editing of GO according to biological relevance to the domain of choice. Creation of a GO subset by clipping is done by removing terms (from all hierarchal levels of the ontology) if they are not functionally relevant to the domain of interest. Terms that are located in levels higher to relevant terms are kept. Thus, biologically irrelevant terms are only removed if they are not parental to relevant terms.

We propose that domain-specific Gene Ontology subsets that contain only terms relevant to specific systems can be created by clipping irrelevant terms from the ontology, while maintaining the ontology consistency i.e. maintaining the relationship between terms as defined in GO (Figure 1). We hypothesize that by considering terms from all levels, better and more comprehensible results for functional analysis of microarray data may be achieved, with minimal loss of resolution. We test this hypothesis by developing a GO subset specializing in the neural and immune systems of human, mouse and rat, and comparing the performance of the resulting clipped GO with that of the full GO and a generic GO-slim. We show that enrichment analysis using the resulting Neural-Immune GO (NIGO) gives better and more interpretable results than the full GO when considering relevant experimental systems, but not in unrelated systems.

## Results

### The Neural/Immune Gene Ontology (NIGO)

**Figure 2:**
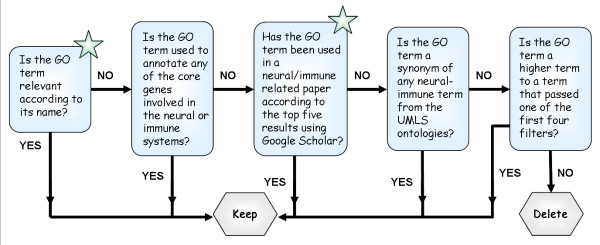
**The five step filtering system**. The five step filtering system used to determine whether any given GO term is biological relevant. Boxes marked with a star are judgment-based, and require a domain expert to consider each term separately. The remaining boxes indicate computational filters.

A neural/immune-specific gene ontology (NIGO) was created by clipping those GO terms that are not associated to any gene in human, rat and mouse, and by clipping terms not found to be relevant to the neural and/or immune domains using a 5-step filtration process (Figure 2, Materials and Methods; see Figure 1 for a definition of the clipping operation). The lists of GO terms removed by each filter can be found in the Supplementary Material (Additional file 1). For the Cellular Component section of GO, only organism-based clipping was performed, since attempts to decide whether terms from this section of GO contribute to the analysis of neural/immune-related datasets were found to be exceptionally difficult and eliminated only a small number of terms. We elected to create a domain specific subset of GO that includes both the neural and immune domains since our interest lies in annotating microarray studies which exploit both these systems.

**Figure 3:**
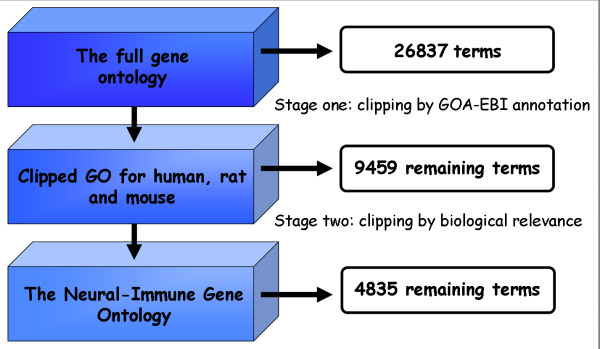
**Overall approach to the creation of NIGO**. The creation of NIGO was done in two main stages: 1. Clipping by relevance to specific organisms. Terms that are not used to annotate genes or gene products in human, rat or mouse (according to the GOA-EBI annotation files) were removed while higher classes of terms kept were also kept to preserve the consistency of the graph. 2. Manually clipping according to biological relevance of the GO terms to the neural/immune systems. A total number of 22,002 terms were deleted from the full GO in order to create NIGO.

The clipping process led to a 5-fold decrease in the number of terms, from 26,837 to 4,835 (Figure 3). Most of the terms were removed through organism-based clipping, which left a total of 9,354 terms, 8,341 that were kept since they were directly associated with one or more human/mouse/rat genes, and 1,013 terms that were kept for being parental to these terms. Organism-based clipping was performed mainly to reduce the burden associated with manual reviewing steps. Clipping according to biological relevance further reduced the ontology by 50%, leaving a total of 4,835 terms, of which 4,175 terms were deemed relevant to the neural/immune systems and 660 terms were parental.

### Comparative analysis of NIGO, GO and GO-slim

NIGO was developed based on the hypothesis that a domain-specific subset of GO will perform better in detecting interesting terms in that domain. In order to test this hypothesis, the performance of NIGO in functional analysis of microarray data was compared to the full Gene Ontology. The performance of each ontology was compared for nine neural and/or immune related microarray data sets and, as a control, three non neural/immune data sets (see Additional file 2). Enrichment analysis was performed for each experiment using the GSEA algorithm [9], chosen for its simple interface that provides the option of uploading user defined gene annotations in the form of gene sets, and for its lack of arbitrary thresholds which need to be separately tuned for each experiment.

The results of this analysis (Tables 1 and 2) show that functional analysis of neural/immune related microarray experiments with NIGO improved the false discovery rate (FDR) values of relevant GO terms in comparison to the full GO with minor loss of relevant terms for the related experiments, but not for neural/immune unrelated experiments.

**Table 1:** Difference in the performance of NIGO, Full GO and GO-slim

A.			
**Gene Ontology Term**	**Full GO**	**NIGO**	**Generic GO slim**

External side of plasma membrane^a^	Not Found	0.14	Not Found

Immune response	Not Found	0.14	Not Found

Antigen processing and presentation of peptide antigen via MHC class I	Not Found	0.170194	Not Found

Peptide antigen binding	Not Found	0.176371	Not Found

MHC class I protein complex	Not Found	0.186667	Not Found

Defense response	Not Found	0.196994	Not Found

Binding^a^	Not Found	0.200462	Not Found

Positive regulation of T cell mediated cytotoxicity	Not Found	0.201567	Not Found

Transport^a^	Not Found	0.214781	Not Found

Mitochondrion^a^	Not Found	0.231303	Not Found

Antigen processing and presentation of exogenous peptide antigen via MHC class I	Not Found	0.235161	Not Found

Protein self-association^†^	0.247254	Not Found	Not Found

Defense response to bacterium	0.239803	0.156774*	Not Found

Response to superoxide	0.24346	0.176879*	Not Found

Plasma membrane	0.246877	0.155097*	0.21

Transporter activity	Not Found	0.187933*	0.21

**B**.			

**Gene Ontology Term**	**Full GO**	**NIGO**	**Generic GO slim**

Integral to membrane^a^	Not Found	0.16	Not Found

Regulation of cell growth	Not Found	0.16	Not Found

Membrane^a^	Not Found	0.16	Not Found

Immune response	Not Found	0.16	Not Found

MHC class I protein complex	Not Found	0.186667	Not Found

Antigen processing & presentation	Not Found	0.224	Not Found

Nucleus	Not Found	0.16*	0.19

**C**.			

**Gene Ontology Term**	**Full GO**	**NIGO**	**Generic GO slim**

Viral capsid	Not Found	0.522253	Not Found

Structural molecule activity^a^	Not Found	0.456971	Not Found

Viral infectious cycle	Not Found	0.406196	Not Found

Viral envelope	Not Found	0.365577	Not Found

Plasma membrane	Not Found	0.553399	Not Found

Protein binding	0.504518	Not Found	Not Found

Binding	0.56242	Not Found	Not Found

Structural molecule activity^a^	Not Found	0.456971*	0.505641

External side of plasma membrane	0.744269	0.508488*	Not Found

(A) Enrichment in genes with up-regulated expression in hippocampus treated with Fluoxetine, based on GEO profile GSE6476. All terms passed FDR > 0.25 and state the FDR values. (B) GEO profile GSE6675. All terms passed FDR < 0.25 and state the FDR values (Up-regulated in Control in comparison to FGF2 treatment). (C) GEO profile GSE6509 All terms passed nomPval < 0.05 and state the FDR values (Up-regulated in LPS Dmso). 'Not Found' indicates that the term was not detected by the analysis. * indicates the best FDR value for that term. ^a ^indicates terms retained in NIGO to keep the consistency of the graph's structure. ^† ^indicates terms that were not included in the NIGO subset.

For three GEO expression profiles, the results of the analysis are described in detail (Table 1). In the analysis of the GSE6476, in which the effect of chronic Fluoxetine treatment on hippocampal gene expression was examined [10], eleven terms passed statistic filtering with NIGO and not with the full GO, out of which 7 were directly related to neural/immune functions or processes (Table 1A). Four of the eleven terms were retained in NIGO due to the graph structure. In addition, NIGO improved FDR scores for terms that were also significant by the full GO (e.g 'Defense response to bacterium'). Only one term that is included in the full GO but not in NIGO passed the statistic cutoffs but this term ('Protein self-association') was functionally irrelevant and contributed very little to the analysis.

For the GEO profile GSE6675, in which astroglial gene expression program elicited by fibroblast growth factor-2 was examined, four terms relevant to neural/immune systems passed statistic filtering with NIGO and not with the full GO (Table 1B). These include the terms 'regulation of cell growth', 'immune response', 'MHC class I protein complex' and 'antigen processing and presentation' which are functionally relevant to the tested system. In addition, two terms passed statistical thresholds with NIGO and were retained due to the graph structure. Furthermore, NIGO improved FDR scores for the term 'nucleus' that was also significant by the generic GO slim subset but not with the full GO. The term 'MHC class I protein complex' received a FDR value of 0.18 when analysis was conducted with NIGO and a FDR value of 0.55 when analysis was conducted with the full GO. This is an example of how without the use of NIGO one would have to raise the cutoff to at least 0.55 in order for this term to appear in the analysis results.

Functional analysis of GSE6509 with NIGO also revealed statistically significant terms that were missed when using the full GO. This experiment involved microarray expression profiling designed to explore the effect of RU486, a synthetic steroid compound (also known as Mifepristone) on LPS-induced gene expression in the CNS [11]. Three relevant terms were revealed by NIGO but not detected with the full GO (Table 1C). These include the terms 'viral envelope', 'viral infectious cycle' and 'viral capsid'. In addition, two terms that were kept in NIGO due to the graph structure rather than their direct relevance to the neural/immune systems were also reported with NIGO but not with the full GO. Intriguingly, two terms that were retained in NIGO (for being parental to relevant terms) passed the statistical cutoffs only for the full GO and not with NIGO. Both of these terms were functionally irrelevant, and both had p-values that are marginally significant for the full GO and generic GO slim (0.025 and 0.0384), and marginally insignificant for NIGO (0.058824 and 0.057692, respectively). This suggests that the difference in analysis stems from the stochastic nature of GSEA, and not from differences in the performance of NIGO and the full-GO. One term passed the statistical criteria both using NIGO and the full GO, in which case the FDR value observed with NIGO was lower than the FDR value obtained with the full GO. The generic GO slim performed very poorly in this analysis, with only one enriched term being noted, namely 'structural molecule activity', which also passed the significance criteria for NIGO with a lower FDR value.

We noticed that some terms, included both in NIGO and the full GO, received higher FDR values when NIGO was used (Table 2), contrary to our expectations from the impact of reducing the ontology size. For example, in the analysis of GSE8788 in which gene expression analysis was conducted using Trib1-deficient macrophages treated with LPS as compared to LPS-treated wild-type macrophages [12], 11 out of 14 terms (that passed statistical cutoff with the full GO and NIGO) had lower FDR values with the full GO. Since NIGO only effects multiple testing correction, we hypothesized that the lack of improvement in FDR values when using NIGO is partially due to the stochastic nature of the GSEA algorithm. To test this hypothesis, the same analysis was repeated three times with each of the ontologies for GSE8788. FDR values were averaged and a comparison of analysis results was performed based on these averaged FDR values. In accordance with our hypothesis, the averaging of FDR values improved the apparent performance of NIGO. Out of the terms that passed the statistical cutoff with NIGO and the full GO, averaged FDR values of 11 terms were lower with NIGO, while analysis with the full GO received lower averaged FDR values for only three terms (for a summary of results with and without averaging of FDR values, see Table 2).

**Table 2:** An overview of the performance of NIGO, full GO and GO-slim in enrichment analysis using GSEA

		Unique to Subset			Lowest FDR Value		
**Experiment**	**Cutoff used**	**Full GO**	**NIGO**	**Generic GO slim**	**Full GO**	**NIGO**	**Generic GO slim**

Neural- or immune-related studies							

GSE6509	P < 0.05	2 (2)	5	0	0	2	0

GSE6675	FDR < 0.25	0	6	0	0	1	0

GSE6476	FDR < 0.25	1 (1)	11	0	0	4	0

GSE3779	P < 0.05	7 (3)	13	1 (1)	0	1	5

GSE8425	P < 0.05	6 (1)	0	0	3	3	1

GSE6690	P < 0.01	2 (2)	0	0	1	8	8

GSE6136	P < 0.05	3	2	0	0	0	1

GSE8788	P < 0.01	6 (6)	0	0	11	3	1

GSE8788*	P < 0.01	6 (6)	0	0	3	11	1

GSE9659	P < 0.01	6 (6)	0	NS^A^	4	28	NS^A^

Non-neural- or immune-related studies (negative controls)							

GSE7407	P < 0.01	5 (5)	0	0	0	1	2

GSE2259	FDR < 0.25	3 (1)	0	1 (1)	1	2	1

GSE8191	P < 0.05	5 (2)	0	0	0	0	0

The enrichment analysis results of GSEA, providing the full GO, NIGO or GO-slim as the sole source of gene sets, are compared for neural/immune-related (top) and unrelated (bottom) studies. A statistical cutoff was chosen for each analysis such that at least five terms were found to be significantly enriched in any of the analyses. Studies are identified via their GEO accession. The number of terms that passed the statistical cutoff inclusively for each subset are provided ('Unique to Subset'), together with the number of terms that are not included in the NIGO subset out of the total number of terms that passed the statistical cutoff only with the full GO or generic slim (given in parentheses). The number of terms that passed the statistical cutoff with NIGO and at least one additional GO subset (the full GO or generic slim) are also shown for each term ('Lowest FDR Value'), with the number of terms with the lowest FDR values for each subset given in separate columns. *To test the effect of stochasticity, the analysis of this profile is based on 3-fold averaged FDR values from three independent GSEA analyses. ^A ^Profile GSE9659 involves rat tissue, and was not analyzed with the mouse GO-slim.

Three non-neural- or immune-related datasets were used to test the performance of NIGO in functional analysis of microarray data in comparison to the full GO and the generic GO slim subset. These datasets included the GEO expression profile, GSE7407, in which gene expression in heart tissue with cardiac specific over-expression of Sirt1 was examined [13], the GEO expression profile, GSE8191, in which the gene expression profile of mammary glands from pregnant mice was compared to that of mammary glands from lactating mice [14], and the GEO expression profile, GSE2259, in which gene expression in testis from sertoli cell-selective androgen receptor knockout mice was examined [15].

In all three of the neural/immune-unrelated datasets, NIGO did not reveal any terms that did not pass the statistical cutoff when conducting the analysis with the full GO or the generic GO slim subset (for an overview of the results, see Table 2; for complete details, see Additional file 3). Terms that passed statistic filtering with the full GO or the slim subset include the terms 'ATP binding', 'mRNA cleavage', 'nucleotide binding', 'integrase activity' and 'positive regulation of mRNA 3'-end processing', none of which are included in the NIGO subset. In addition, multiple terms with marginal significance were detected with the full GO but not NIGO due to the stochastic nature of GSEA (e.g. 'proteolysis', 'cytoplasm', 'caspase activation' and 'metal ion binding'). Due to its reduced size, those terms that were identified with NIGO and the full GO and/or the generic GO slim often received lower FDR values in NIGO.

These results were further supported by analyzing the same expression profiles with Ontologizer [16], which uses a modified Fisher Exact Test, to test deterministically for over-representation of each GO term, given two groups of genes, and using the Benjamini-Hochberg method to control for the false discovery rate. Most terms that passed the statistical threshold for both the full GO and NIGO had lower adjusted p-values when analysis was conducted with NIGO (Table 3 and Additional file 4). However, in the case of two non-neural- or immune datasets, some terms received lower adjusted p-values with the full GO than with NIGO. Careful examination of the data suggests that this is the result of the multiple testing correction method, as the distribution of p-values shifts when terms are omitted, leading to differences in the adjusted p-value assigned to the remaining terms. Thus, Ontologizer gave results that partially reproduce the phenomena observed with GSEA, both in terms of improved sensitivity of the analysis with NIGO over the full GO and in the occasional lower p-value estimate (after multiple testing correction) for the full GO when non-neural/immune datasets are analyzed.

**Table 3:** An overview of the performance of NIGO, full GO and GO-slim in enrichment analysis using the Fisher Exact Test

		Unique to Subset			Lowest FDR Value		
Experiment	Cutoff used	Full GO	NIGO	Generic GO slim	Full GO	NIGO	Generic GO slim
Neural- or immune-related studies							
GSE6509	P < 0.0001	1 (1)	1	0	0	22	2
GSE6675	P < 0.05	1 (1)	0	11 (1)	0	2	0

GSE6476	P < 0.0001	0	2	2	0	15	3

GSE3779	P < 0.1	0	0	0	0	2	0

GSE8425	P < 0.1	0	0	0	0	0	0

GSE6690	P < 0.001	0	2	0	0	9	1

GSE6136	P < 0.000001	0	1	0	0	37	7

GSE8788	P < 0.01	0	2	0	0	4	1

GSE9659	P < 0.001	0	3	NS^A^	0	33	NS^A^

Non-neural- or immune-related studies (negative controls)							

GSE7407	P < 0.0000000001	33 (30)	0	0	9	0	0

GSE2259	P < 0.1	63 (35)	0	2	8	8	7

GSE8191	P < 0.001	6 (6)	7	6 (2)	0	16	3

Enrichment analysis results from Ontologizer, providing the full GO, NIGO or GO-slim as the GO subset to be used are compared for neural/immune-related (top) and unrelated (bottom) studies. Studies are identified via their GEO accession. The number of terms that passed the statistical cutoff inclusively for each subset are provided ('Unique to Subset'), together with the number of terms that are not included in the NIGO subset out of the total number of terms that passed the statistical cutoff only with the full GO or generic slim (given in parentheses). The number of terms that passed the statistical cutoff with NIGO and at least one additional GO subset (the full GO or generic slim) are also shown for each term ('Lowest P-Value'), with the number of terms with the lowest P-values for each subset given in separate columns. ^A ^Profile GSE9659 involves rat tissue, and was not analyzed with the mouse GO-slim.

These results, together with the analysis of five additional neural/immune-related experiments not described in Table 1 (namely profiles GSE3779, GSE8425, GSE6690, GSE6136 and GSE9659) are summarized in Table 2 (see Additional file 3 for complete results).

## Discussion

We present here an approach for generating domain-specific subsets of GO, via an editing method we call 'clipping'. We show that the use of a clipped subset of GO can improve functional analysis of microarray data relevant only to the domain of the clipped ontology. We present NIGO, a clipped subset of GO directed at nervous and immune systems. Evidence that the immune system affects neuronal processes, such as neuronal maintenance and repair, is accumulating. It was recently shown, for example, that mice lacking both T- and B-cell populations (Severe Combined Immune Deficiency, SCID) show impairment in neural precursor cell proliferation and differentiation into mature neurons [17]. We thus chose to create a neural and immune subset of the Gene Ontology since we were specifically interested in annotating microarray experiments which link the two systems. Even though the design of the subset is aimed for the annotation of such experiments, NIGO is also useful for the annotation of expression studies which investigate only one of these two biological systems. We show that NIGO outperforms the full GO or a generic GO-slim in finding relevant terms that are enriched in genes with varied expression in these systems. NIGO revealed GO terms, from all hierarchical levels of the ontology, that were relevant to the experimental system used to create the microarray data and that did not pass the statistical cutoff when conducting the analysis using the full GO. In addition, NIGO improved the statistical scores assigned to many neural/immune-relevant GO terms that passed the statistical cutoff for the full GO.

It is important to stress the fundamental difference between a clipped subset of GO and a slimmed subset. While a slimmed subset will also achieve improvement of statistical scores assigned to the enriched terms, theses terms will be general top-level terms that will reveal a lot about the nature of the biological differences between two sets of samples, but little regarding the specific responses. The power that lies in a clipped subset of GO is not only in improvement of statistical scores but also in enrichment of terms from all hierarchical levels which reveals more about the biology underlying the study. In this work, we used a generic slim subset which contains 152 top-level GO terms. While a neural/immune specific GO slim could have performed better than a generic slim, it would most likely not include specific terms, such as 'Antigen processing and presentation of exogenous peptide antigen via MHC class I' and 'Positive regulation of T cell mediated cytotoxicity' which are located 6 and 7 steps away from the root, respectively. Such terms proved to be important for the interpretation of a relevant microarray dataset (see Table 1) and thus demonstrate how a clipped subset of GO could outperform even a specific slimmed subset.

In one study, the use of NIGO actually allows the formulation of hypotheses that are otherwise missed when using the full GO. In studying the effect of chronic Fluoxetine treatment on hippocampal gene expression, Miller *et. al. *compared expression patterns in the hippocampi of mice with or without treatment with the antidepressant, Fluoxetine, for 21 days [10]. NIGO revealed several immune-related terms that were not identified with the full GO. These include GO terms such as 'antigen processing and presentation of peptide antigen via MHC class I', 'MHC class I protein complex', 'positive regulation of T cell mediated cytotoxicity', and 'defense response' Furthermore, this group of terms was not found to be significantly enriched in the original analysis of this dataset.

The hypothesis that can be derived from this finding, namely that treatment with Fluoxetine alters immune-related processes in the brain via the MHC-class I pathway, is in agreement with previous knowledge. Chronic stress and depression are widely known to down-regulate the immune system and several lines of evidence indicate that some antidepressants can reverse this impairment by producing various immunomodulatory effects [18-20]. Interestingly, it was shown that uptake of serotonin 5-hydroxytryptamine (5-HT) is impaired by Fluoxetine, a process which may interfere with mechanisms of immune regulation [21]. Moreover, rats treated with Fluoxetine demonstrated reduced CD4+ cell number, increased number of CD8+ cells and elevated levels of cytokines such as IL4 and IL2 in vitro [22]. The NIGO-based finding thus coincides with the observed increase of CD8+ cells since the term 'positive regulation of T cell mediated cytotoxicity' was found by NIGO to be enriched in the dataset. Furthermore, most cytotoxic T cells express T-cell receptors (TCRs) that recognize a specific antigenic peptide bound to class I MHC molecules. Accordingly, GO terms related to MHC class I antigen presentation were also found by NIGO to be enriched in this dataset. This line of evidence suggests that Fluoxetine, and possibly other antidepressants, exert their effects, at least partially, via modulation of CD8 T cell's activity. These results further demonstrate that analysis with NIGO can enhance interpretation of functional analysis results produced for relevant microarray datasets.

In the analysis of the GSE6509 expression dataset, three relevant terms passed the statistical cutoff with NIGO but not with the full GO. These terms, 'viral envelope', 'viral infectious cycle' and 'viral capsid' are all terms related to mouse genes involved in viral infection. It was previously shown that for this dataset, clustering of the GO nodes revealed that the GO term 'response to virus' was enriched in genes down-regulated by LPS treatment [11]. Several lines of evidence show a connection between Mifepristone and viral infection. For example, it was shown that Mifepristone can increase target cell sensitivity to retroviral infection [23]. Though the analysis of this dataset with NIGO did not reveal any new biological knowledge, detecting and interpreting the 'response to virus' related terms was made much easier.

In several cases, terms were detected by the full GO but did not pass the same cutoff when conducting functional analysis with NIGO, even though these terms were included in the NIGO subset. Such terms received FDR or p-values that were very close (but larger) than the cutoff values used. This is partially explained by the stochastic nature of the GSEA algorithm. Indeed, for one set (GSE8788), we compared the raw results with averaged FDR values. Averaging dramatically decreased the number of such terms. Furthermore, we conducted a similar functional analysis using the Fisher Exact Test and found that while this method included no stochastic element, several of terms that were found by both the full GO and NIGO (in the analysis of two non-neural/immune related datasets) had lower adjusted p-values when conducting the analysis with the full GO (see the Results section).

Out of nine neural/immune-related expression datasets used to evaluate the performance of NIGO in comparison to the full GO, NIGO revealed terms that did not pass statistical cutoff with the full GO for five. For two of the four datasets in which NIGO did not reveal new terms, this ontology improved the FDR values for over half of the terms that passed the statistical cutoff with the full GO. Hence, for approximately 77% of the datasets used, NIGO outperformed the full GO either by revealing new terms or improving FDR values for more than 50% of the terms that passed the statistical cutoff with both ontologies. For neural- or immune unrelated microarray studies, on the other hand, NIGO did not outperform the full GO or the generic GO slim. These results are well in agreement with the design and purpose of NIGO.

**Figure 4:**
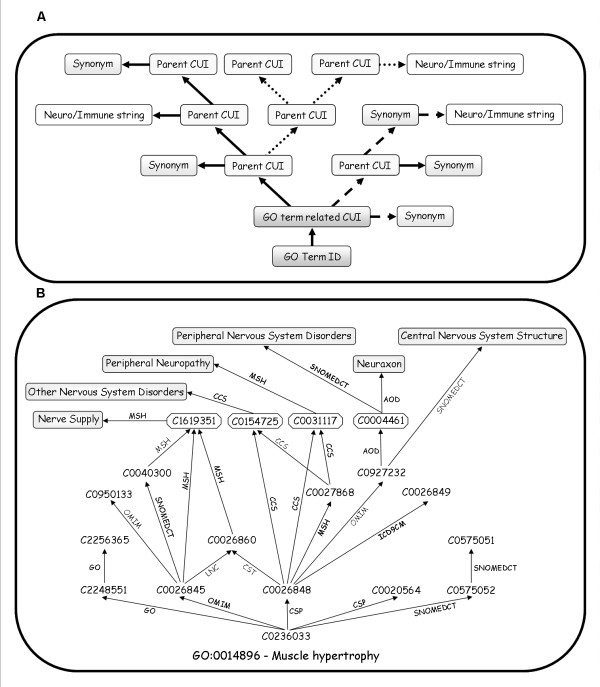
**The UMLStermFinder**. (A) The underlying algorithms. A GO term is associated with a Unique Concept Identifier (CUI) using the UMLS classes file (MRSAT). The UMLS relations file (MRREL) is then used to identify CUI terms that are parental to the input GO terms, recursively traversing the DAG up to 3 levels up. All the aliases to any of these terms are also extracted. Each CUI found in the resulting sub-graph is associated with their corresponding strings, which are searched for any of the following: 'immune', 'neuro', 'inflam', 'brain', 'lymph' or 'nerve'. The outputs of the algorithm are the terms that contain these strings. Arrows of different types denote different sources from which relationships were asserted. (B) An example of how UMLStermFinder works. Using the GO term 'muscle hypertrophy' (GO:0014896) to search the UMLS ontologies, the corresponding concept identifier is found and used to find the concept's parents and synonyms (framed CUIs) of the initial concept and its parents. After all concepts are collected, they are searched for neural and/or immune-related string content (grey boxes). The UMLStermFinder finds concepts from several ontologies; by each arrow in the figure appears the name of the source ontology from which the relationship was asserted. A Perl script implementing the UMLStermFinder algorithm is freely available as Additional file 7.

Alternative approaches may lead to the creation of domain-specific subsets from the GO. One approach involves the selection of terms that are descendant of the GO term or terms that are most pertinent to that domain. In the case of NIGO, this would mean choosing all those terms that are direct descendants of the terms 'immune system process' (GO:0002376) or 'neurological system process' (GO: 0050877). We believe that this approach would lead to many relevant terms being omitted, since not all pertinent terms are necessarily defined in GO as a neural or immune system process/function. For example, the term 'muscle hypertrophy' (GO:0014896) is not defined in GO as a neurological system process but was found to be linked to the neurological system by the UMLStermFinder (Figure 4B). It is highly likely that not all of the information linking biological processes to these systems have been incorporated into the Gene Ontology. It is our opinion that by adding knowledge from the literature and from other biomedical ontologies (included in UMLS), terms that are not directly associated with these biological systems can still be included in NIGO, or any other domain-specific subset. Another possible approach is to create a domain-specific slim. While this approach will probably create a highly compact ontology, setting some threshold of abstraction, which is the declared purpose of GO slimming, would necessary lead to loss of information. In this study, for example, it is hard to imagine a GO slim that would go so far down the GO graph as to include the terms 'Antigen processing and presentation of exogenous peptide antigen via MHC class I' and 'Positive regulation of T cell mediated cytotoxicity' without defeating the purpose of the slimming process. Yet these two terms were found to be enriched in GSE6476, and are crucial for generating a hypothesis based on the expression profile. This shows that GO slims may be complemented by small, yet fully detailed domain-specific subsets of GO.

NIGO is obviously not a perfect representation of all knowledge related to neural/immune-related gene function. Due to the massive use of human-based curation of GO terms used for the production of NIGO, wrong judgment calls have most likely lead to some erroneous inclusion or exclusion of terms. The use of the core gene set (filter 2) may lead to errors of commission due to the multifunctional nature of genes. Thus, the use of the subset in analyzing high-throughput experiments may lead to some loss of information and may include irrelevant terms. Error of commission only leads to a slight degradation of the statistical power of analysis as long as the fraction of falsely included terms is small. Since the results must be interpreted by someone versed in the domain to be useful, the inclusion of a small fraction of non-specific terms would do little to degrade the usefulness of the results. Errors of omission, on the other hand, may more significantly degrade the results. In theory, it is sufficient that one important term be missing to blind the interpreter from seeing the true biological meaning of the results. Nevertheless, our analysis of actual microarray data demonstrates that even in its current form NIGO allowed interesting enrichments to be identified that were otherwise missed. However, to overcome the impact of errors of omission, at least in part, we recommend that in addition to using NIGO (or other subsets generated in a similar fashion), one should conduct a parallel analysis using the full GO. This will ensure that the domain-specific GO subsets could do no harm - the interpretation of the results using both full and clipped GO cannot be less informative than interpreting the full GO alone.

The approach we present here for constructing domain-specific GO clipping can be applied to other fields. It is possible, for example, to divide NIGO into immune-specific and neural-specific sets. It should also be possible to generate specific subsets that are relevant to other domains, although significant work is involved in the process, especially in the step involving a domain expert's review of a significant fraction of all GO terms. It is possible that this step can be largely replaced by automatic procedures, or that the pipeline described here can be improved to reduce the number of decisions that require an expert's opinion. Furthermore, the five-step filter used to decide upon NIGO inclusion/exclusion could be altered to include more steps, such as searches of other knowledge sources. Improved automation, or alternatively a major community effort, could lead to the creation of a library of domain-specific clipped GO subsets, which could, in turn, enhance the interpretability of many microarray experiments. Improved automation could, in principle, be obtained by reversing the logic of our NIGO evaluation, domain-related GO terms can be picked by examining the GO terms that are associated with expression profiles deemed by domain experts to be related to some domain.

Another challenge in developing high quality GO subsets is their maintenance. The Gene Ontology is constantly growing, with new terms and annotations to genes being regularly added. It is thus important to continually update GO subsets, such as NIGO. NIGO could be updated by periodically reviewing new terms and annotations that have been added to the GO, subjecting them to the filter system developed to find relevance of terms to the neural/immune systems and adding the relevant terms, along with their parental terms, to the subset. Assuming no dramatic increase in the rate of growth of the GO, this can be achieved with modest effort as the number of new terms is much smaller than the number of existing terms.

Further research into the automation of domain-specific ontology clipping and/or community efforts may lead to the emergence of multiple domain-specific derivates of GO that will improve the interpretability of high-throughput gene-related analyses.

## Conclusions

We developed NIGO, a clipped subset of the Gene Ontology directed at the neuronal and immune systems. NIGO was validated by showing that it indeed improves the functional analysis results of neural/immune related expression profiles. Moreover, in the analysis of at least one dataset NIGO allowed generation of a hypothesis which would otherwise have been missed. We thus propose that clipped domain-specific GO subsets can produce clearer functional analysis results and help generate meaningful hypotheses.

## Methods

### Overall approach

NIGO was created by removing terms not associated with any gene in human, mouse and rat, and choosing neural/immune relevant terms using a five step filtering system. The performance of the resulting NIGO was analyzed in comparison to the full GO and a generic GO slim by comparing their performance in functional analysis of relevant and non-relevant microarray data.

### Data and tools

Ontology source files (the full GO and the generic mouse slim subset of GO) were obtained from the GO consortium web site [24] in the OWL format [25] (October 2008).

The slim subset contains 152 high level GO terms listed at http://bioinfo.bgu.ac.il/rubin/supplementary/NIGO/Supplementary.html.

The ontologies were clipped using the Protégé 4.0 beta OWL editor [26].

For by-species filtering, annotation files for human, rat and mouse were downloaded (October 2008) from GOA-EBI [27]. Association files used for GSEA analysis were generated based on the GOA-EBI annotation files and in the format required by GSEA. In this format, each row represents a GO term. The first column contains the GO term ID, the second column contains a description (or NA) and the rest of the columns contain the genes that GO term is used to annotate. For NIGO, the association file contained only terms included in the subset. These files can be found at http://bioinfo.bgu.ac.il/rubin/supplementary/NIGO/Supplementary.html. The mapping between these terms and genes was kept identical to the ones in the GOA-EBI annotation files. For UMLS-based filtering, the UMLS version 2008AB files were used [28]. Microarray data analysis and functional analysis were conducted using the GenePattern [29], GSEA [9] (release 2.5) web servers, and Ontologizer [16] as follows: (1) for each study, raw data (.CEL files) were downloaded from GEO [30]; (2) expression files (.gct files) were created using the Gene Pattern Expression File Creator module; (3) where necessary (i.e. for expression files GSE6509, GSE6675, GSE6476, GSE7407, GSE8788 and GSE9659), preprocessing was applied using the Gene Pattern Preprocess Dataset module. (4) Functional analysis was conducted using the GSEA module. GSEA was run three times for each dataset, using a different GO version for each run. For the full GO, we used the organism-specific GO subset. In the analysis of GSE8788, GSEA was run three times for each of the three ontologies and FDR values were averaged over the three runs. (5) Differentially-expressed genes were found using the Gene Pattern ComparativeMarkerSelection module. Cutoff values for selection of genes were chosen such that for each dataset, at least 150 differentially-expressed genes were found in at least one sample set. (6) Functional analysis was conducted (April 2010) using Ontologizer. Term-for-term analysis was conducted and the Benjamini-Hochberg method was selected for multiple test correction. Cutoff values for significant GO terms were selected based on the results obtained with the full GO but were never higher than 0.1. For analysis, NIGO was defined as a subset in the full GO file (OBO format) and all GO terms included in NIGO were tagged as belonging to this subset (available from: http://bioinfo.bgu.ac.il/rubin/supplementary/NIGO/Supplementary.html). The same strategy was performed on the generic mouse slim subset. For a complete description of the parameters used to run each of the GenePattern modules, see Additional file 5.

### The 5 step filter system

Each term was subjected to testing using a five-step filter. In each step, terms were selected based on their relevance to the neural/immune systems. Only terms that were not selected were passed forward to the next step (Figure 2).

1. Filtration by name: Based on a domain expert's knowledge, terms were selected if their name indicated their relevance (for example, the terms 'neuron migration' or 'establishment of T cell polarity' would pass this filter and will be included in NIGO). Terms were selected for inclusion if they seemed even remotely relevant to the neural- or immune domains. This step involved manual evaluation of thousands of GO term names. Note that this step is subjective, making NIGO biased towards the opinion of the experts who evaluated the terms.

2. Association with a core gene list: A subset of genes described in the literature as neurological or immunological markers was compiled (see below). These genes are routinely used in our lab as biomarkers for neurological and immunological processes. We note that selecting these genes was partially subjective and based on our past experience. Terms used to annotate at least one gene from this list were flagged for inclusion in NIGO. The genes used in the core group were PCNA, PVALB, Tuj1, Calb1, Calb2, DCX, DPYSL2, HH3, Eno2, GFAP, MKI67, MSI1, Nes, NeuN, Map2, NT3, P75 (NGFR) for neural processes and STAT1, STAT3, SOCS1, SOCS2, IGF1, IFNgR, IL1b, IL6, BDNF, CNTF, IFNg, TNF, IL4 for immunological processes.

3. Google Scholar search: The term name, when used to search with Google Scholar, returned a neural- or immune-related paper in the top 5 results. Searches were performed (October - November 2008) by seeking exact matches to the term name and adding at least one key word from the following list: 'neuro', 'immune', 'inflam', 'lymph', 'nerve', and 'brain'. This filter was applied liberally, i.e. terms were selected if search results were deemed even remotely related to the neural- or immune domains.

4. UMLS-based filtration: terms are flagged as positive results using the UMLStermFinder (see below).

5. Ontological integrity: Terms were selected to be included in NIGO if they were parental (i.e. a super-class by means of an 'is_a' or 'Part_of' relationship) to any term that was flagged as positive by any of the other filters. The 'is_a' relations were traversed up to the root of the graph (in such a way that for each term included in NIGO the direct and indirect 'is_a' parents were included) and part_of relations were only used to include directly related GO terms. Links via the 'regulates', 'negatively regulates' and 'positively regulates' were not traversed.

### UMLStermFinder

The UMLStermFinder is a tool developed for this study for federated searches across the multiple ontologies included in the UMLS. It tests the relevance of a GO term to the neural- or immune systems by traversing the UMLS, seeking neural/immune-related terms that are connected, directly or indirectly, to an input term (Figure 4). The connections of UMLS concepts to the tested GO term are provided by UMLS and defined within the UMLS data files.

### Microarray data sets

All microarray data sets were downloaded from GEO at NCBI [30]. The GEO sets used in this study are described in Additional file 2.

### Availability

NIGO is freely available as Additional file 6 and for download from: http://bioinfo.bgu.ac.il/rubin/supplementary/NIGO/Supplementary.html as flat-file, or in the OWL and Open Biomedical Ontologies (OBO) [31] formats.

All supplementary material is freely available for download from: http://bioinfo.bgu.ac.il/rubin/supplementary/NIGO/Supplementary.html

## Authors' contributions

NG, AM and ER conceived the idea and designed the research; NG and ER conducted the research and wrote the article. All authors have read and approved the final manuscript.

## Supplementary Material

Additional file 1 **Filtered GO terms**. This file contains all the terms that were tested using the five-step filter, by which step of the filter they were flagged and which of the terms were included or excluded from NIGO. Click here for file

Additional file 2 **Microarray Datasets for Comparative analysis of NIGO, GO and GO-slim**. This file contains a summary of the microarray datasets downloaded from GEO at NCBI and used to test the performance of NIGO. Click here for file

Additional file 3 **GSEA analysis results**. This file contains a summary of the GSEA analysis results for each of the microarray studies used to test the performance of NIGO, GO and GO-slim. Click here for file

Additional file 4 **Ontologizer analysis results**. This file contains a summary of the Ontologizer analysis results for each of the microarray studies used to test the performance of NIGO, GO and GO-slim. Click here for file

Additional file 5 **Parameters Used to Run GenePattern Modules**. This file contains the parameters used to run GenePattern modules (Parameters not mentioned here were left as default) Click here for file

Additional file 6 **NIGO**. This file contains the NIGO ontology in the OWL format. Click here for file

Additional file 7 **MLStermFinder**. This file contains the UMLStermFinder script implemented in Perl. Click here for file
