# Supplementary material for: The Neural/Immune Gene Ontology: clipping the Gene Ontology for neurological and immunological systems
Source: BMC Bioinformatics. 2010 Sep 12;11:458. doi: 10.1186/1471-2105-11-458 (PMC2949890; doi:10.1186/1471-2105-11-458)
Supplement: Additional file 2 — Microarray Datasets for Comparative analysis of NIGO, GO and GO-slim. This file contains a summary of the microarray datasets downloaded from GEO at NCBI and used to test the performance of NIGO. [file 1471-2105-11-458-S2.DOC]

Microarray Datasets for Comparative analysis of NIGO, GO and GO-slim

Microarray datasets downloaded from GEO at NCBI and used to test the performance of NIGO.

| **Neural/immune related** | | |
| --- | --- | --- |
| **Experiment name** | **GEO accession** | **Experiment description** |
| Gene Profile of RU486 effect on LPS induced gene expression in CNS [Mus musculus] | GSE6509 | Gene expression microarray analysis was conducted using brain samples from mice treated with bacterial lipopolysaccharide (LPS) and glucocorticoid-receptor antagonist (RU486 – mifepristone) in comparison to samples taken from mice treated with LPS alone. |
| Astroglial gene expression program elicited by fibroblast growth factor-2 [Mus musculus] | GSE6675 | Gene expression microarray analysis was conducted using astroglial cells treated with FGF2 in comparison to control samples. |
| Effect of chronic fluoxetine treatment on hippocampal gene expression [Mus musculus] | GSE6476 | Gene expression microarray analysis was conducted using hippocampus of mice treated with fluoxetine in comparison to control samples. |
| p107 knockout vs. wild type neurospheres [mus musculus] | GSE3779 | Gene expression microarray analysis was conducted using p107-/- neurospheres (cultured from the brain of E13.5 embroys) in comparison to wild type neurospheres. |
| Ras-GRF1 deficiency effect on the hippocampus [Mus musculus] | GSE8425 | Gene expression microarray analysis was conducted using laser capture microdissected hippocampi of mutants lacking Ras-GRF1 in comparison to wild type. |
| ANA-1 macrophages infected with Chlamydia pneumoniae [Mus musculus] | GSE6690 | Gene expression microarray analysis was conducted using mouse macrophages (ANA-1 cells) that were infected in vitro with C. pneumoniae with a M.O.I. of 10 in comparison to untreated macrophages. |
| Expression data from murine BRD2-mediated lymphomas [Mus musculus] | GSE6136 | Gene expression microarray analysis was conducted using resting B-cells that are E-mu-BRD2 transgenic (aggressive lymphoma) in comparison to resting wild-type B-cells. |
| Comparison of gene expression pattern between Wild-type and Trib1-deficient mice [Mus musculus] | GSE8788 | Gene expression microarray analysis was conducted using Trib1-deficient macrophages treated with LPS in comparison to wild-type macrophages treated with LPS. |
| **Experiment name** | **GEO accession** | **Experiment description** |
| Expression data for rat CNS mixed glial cultures treated with cytokines [Rattus norvegicus] | GSE9659 | Gene expression microarray analysis was conducted using mixed CNS glia cultures from newborn rat brain were that were treated for 6 hours with a cytokine mixture representative of Th1 cytokines in comparison to mixed CNS glia cultures that were not treated. |
| **Neural/immune unrelated** | | |
| **Experiment name** | **GEO accession** | **Experiment description** |
| Regulation of Gene Expression by Sirt1 in the heart [Mus musculus] | GSE7407 | Gene expression microarray analysis was conducted using the heart from transgenic mice with cardiac specific overexpression of Sirt1 (Tg-Sirt1) at 3 months of age in comparison to non-transgenic (NTg) control littermates. |
| Key stages in mammary gland development [mus musculus] | GSE8191 | Gene expression microarray analysis was conducted using mammary glands from pregnant mice in comparison to mammary glands from lactating mice. |
| Androgen effect on spermatogenesis: time course [Mus musculus] | GSE2259 | Gene expression microarray analysis was conducted using testis from sertoli cell-selective androgen receptor knockout (SCARKO) on postnatal d 10 in comparison to littermate controls. |
