# Supplementary material for: The Neural/Immune Gene Ontology: clipping the Gene Ontology for neurological and immunological systems
Source: BMC Bioinformatics. 2010 Sep 12;11:458. doi: 10.1186/1471-2105-11-458 (PMC2949890; doi:10.1186/1471-2105-11-458)
Supplement: Additional file 5 — Parameters Used to Run GenePattern Modules. This file contains the parameters used to run GenePattern modules (Parameters not mentioned here were left as default) [file 1471-2105-11-458-S5.DOC]

Parameters Used to Run GenePattern Modules

Parameters used to run GenePattern modules (Parameters not mentioned here were left as default)

**ExpressionFileCreator (Gene Pattern):**

Method – RMA

Background correct and quantile normalization were used

Normalization method – median scaling.

**PreprocessDataset module:**

Filter flag - filter

Preprocessing flag – no discretize or normalization

Minchange – 1

Min delta – 100

Threshold – 20

Ceiling – 20000

Max sigma binning – 1

Prob thres – 1

Num excl – 0

Log base 2 – no.

**Gene Set Enrichment Analysis (GSEA):**

Gene sets database – c3.mir v2.5

Number of permutations – 100

Collapse dataset – true

Permutation type – phenotype

Scoring scheme – weighted

Metric for ranking genes- diff of means

Max gene set size – 500

Min gene set size – 1

**Comparative Marker Selection:**

Test direction – 2 sided

Test statistic – T test

Number of permutations - 1000

Phenotype test – all pairs

*Parameters not mentioned here were left as default
